# Rethinking the egg

**DOI:** 10.1093/nsr/nwad288

**Published:** 2023-11-20

**Authors:** Michael J Benton

**Affiliations:** School of Earth Sciences, University of Bristol, UK

Our image of early dinosaur eggs must change. Early dinosaur eggs are portrayed as soft-shelled, like those of modern lizards or snakes [1], and yet all the evidence is that early dinosaurs at least laid leathery-shelled eggs, more like some turtle eggs such as *Chelydra serpentina* and *Pseudemys nelsoni*. This matters because the protective egg is key to the success of dinosaurs, as well as modern reptiles and birds. Han *et al.* [2] present a detailed account of the nest of an Early Jurassic sauropodomorph and the eggs are leathery, not soft-shelled or hard-shelled.

A key evolutionary transition is marked by the amniotic egg, which enabled the first reptiles to lay their eggs safely on land without risk of their drying out, and this marks the success of all Amniota (=reptiles + birds + mammals). Until recently, the first amniotic egg was assumed to have been hard-shelled because that is what we see in modern birds, crocodilians, turtles and monotreme mammals. The hard eggshell is mineralized, comprising crystalline units of calcium carbonate.

However, a leathery egg is typical of some turtles and mammals [3,4]. For example, the egg of monotremes (echidnas, platypus) is mineralized and white, like a hen's egg, but in fact the eggshell is leathery with only a very thin layer of mineralized calcium carbonate [3]. Among turtles, sea turtles and some tortoises (e.g. *C. serpentina* and *P. nelsoni*) also lay leathery eggs with a thin calcareous layer [4,5]. Most evidence confirms that the eggs of early dinosaurs shared similar features with these leathery eggs.

The new discovery [2] includes three dinosaur skeletons, five nests, some eggs and embryos, all from the Ziliujing Formation of Guizhou Province, dated as 199–193 Ma. The dinosaur, *Qianlong*, is about 6 m long and the embryos only 15 cm. The babies were quadrupedal, as has been seen in other dinosaurs, and grew up to be bipedal, providing an interesting parallel in ontogeny to phylogeny, where dinosaur ancestors were quadrupeds and dinosaurs themselves were bipedal from the start.

The eggs are 11.5 cm long and 9.4 cm wide, and they have a thin calcareous layer, but they are interpreted as leathery because this mineralized layer is very thin (about 0.2 mm, not 1 mm, as expected from an egg of this size), the outer surface of the egg is rough in texture, the overall egg shape is irregular, and the calcareous layer breaks up into tiny pieces when damaged. The difference between a hard eggshell and a leathery eggshell is not simply the presence or absence of mineralization, but the thickness and hence its ability to support the egg or not.

**Figure 1.**
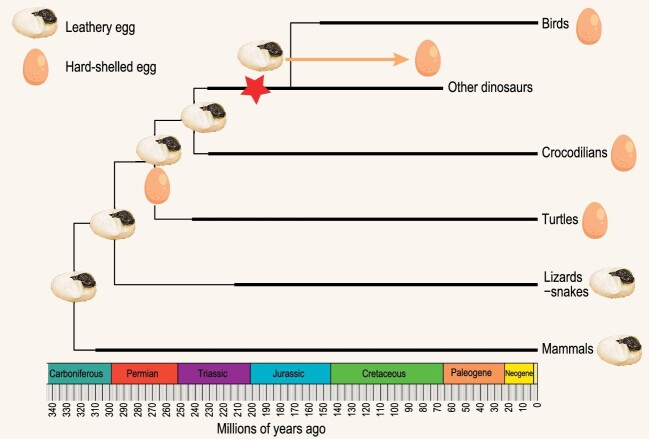
Evolution of egg shells, showing probable ancestral states. Position of the new fossil marked with red star.

The ancestral states analysis (Fig. 1) shows that the ancestral dinosaurs had leathery eggs, and this is probably true also for the first diapsid, and for all amniotes, as argued also by other authors [6,7]. So, we must change our perceptions of the function of the amniote egg in the first half of its evolution. Early reptiles laid an egg with a shell, but the shell was probably leathery, and the same was true for the first dinosaurs. Perhaps dinosaurs, crocodilians and turtles all originally laid leathery eggs and then independently evolved an eggshell with a thick calcareous layer at least three or four times—once on the dinosaurian line to birds, at least twice in other dinosaur lines and perhaps independently also in crocodilians and in turtles. We can no longer picture these early reptiles with bird-like eggs in their nests!
